# Geolocated Lightning Network topology snapshots: A dataset covering 2019–2023

**DOI:** 10.1038/s41597-025-06413-7

**Published:** 2025-12-10

**Authors:** Danila Valko, Jorge Marx Gómez

**Affiliations:** 1 Carl von Ossietzky Universität Oldenburg, Department für Informatik, Ammerländer Heerstraße 114-118, 26129 Oldenburg, Germany; 2 OFFIS – Institute for Information Technology, Escherweg 2, 26121 Oldenburg, Germany

**Keywords:** Research data, Communication, Geography

## Abstract

The Lightning Network (LN) is the most widely adopted second-layer solution for Bitcoin, enabling fast, low-cost transactions through a decentralized payment channel network. Despite its growing importance and the increasing interest from researchers across disciplines, progress in LN research is often impeded by limited access to structured, validated, and reproducible network data. In this paper, we present a curated collection of LN network snapshots spanning from January 2019 to July 2023, reconstructed from publicly available gossip message archives. We apply rigorous consistency checks, and enrich node metadata with city-level geolocation data derived from public IP addresses. The resulting dataset captures the temporal and spatial evolution of the LN, addresses a critical research gap, and provides a reproducible foundation for future empirical studies on network structure and dynamics – accessible not only to the computer science community but also to researchers in cryptocurrency and economics.

## Background & Summary

The Lightning Network (LN) is the most prominent payment channel network and a leading second-layer solution built on top of Bitcoin. It forms a large, sparse, and globally distributed instant payment system. As of October 15, 2025, the LN consists of approximately 12,632 active nodes and 43,758 payment channels, collectively holding around 4,053 BTC (https://1ml.com/statistics). For a broader technical overview, readers are referred to existing reviews on blockchain-based payment channel networks, their challenges, and recent advances^1,2^.

The LN is continually evolving and serves as a dynamic testbed for the future of payment channel infrastructure. Over the past decade, it has attracted substantial attention from researchers in engineering, computer science, network science, and economics^1^. Recent studies have explored diverse topics such as network topology and infrastructure^3–5^, security mechanisms^6–8^, routing protocols^9–11^, profitability^12,13^, and the broader implications for the Bitcoin ecosystem^14,15^.

Despite this growing interest, meaningful research is often hindered by the difficulty of acquiring, storing, and processing relevant network data. In many cases, researchers must first develop their own data collection infrastructure before addressing core research questions. Consequently, fewer than 20% of studies utilize real-world network data, and less than 5% publicly share the data they collect^2^. As a result, most existing analyses rely on self-gathered or reconstructed snapshots derived from raw data sources that are often non-public, extremely fragmented, unvalidated, and non-reproducible.

These challenges highlight the critical need for unified and validated benchmark network topology snapshots constructed from independent sources in a reproducible manner. To address this gap, we provide a curated dataset of 336 geolocated LN topology snapshots, spanning the years 2019 to 2023, designed to improve data accessibility for researchers lacking the infrastructure or expertise to collect or reconstruct such snapshots themselves. This collection aims to bridge the technical and data divide, enabling more rigorous and reproducible future research. Furthermore, our reconstructed network snapshots are cross-validated against independent statistics to establish a robust and reliable benchmark.

## Methods

This section outlines the dataset generation process, which involves acquiring network message data, reconstructing network graphs, performing data cleaning and consistency checks, and node geolocation. All data processing routines were implemented in Python (available at https://github.com/ellariel/ln-data-preparation).

### Stage 1: Data acquisition

In the LN, nodes must be aware of the network topology to route payments efficiently. To enable this, nodes exchange *gossip messages* that announce the creation or modification of nodes and channels. These messages follow the Basics of Lightning Technology (BOLT, https://github.com/lightningnetwork/lightning-rfc/) specifications and consist of three primary types^3^:

- node_announcement – Advertises a node’s metadata beyond its public key, including its IP address, alias, color, timestamp, and any supported higher-layer protocols.
- channel_announcement – Announces the creation of a payment channel between two nodes, specifying the short channel ID and the public keys of both participants.
- channel_update – Describes the directional properties of a channel. Because LN channels are bi-directional, each participant must broadcast a separate update for their side.

Since gossip messages are not stored on the blockchain and no official message archive exists, researchers typically collect them independently, making historical network reconstructions and reproducibility difficult. Fortunately, a publicly available archive maintained by C. Decker and collaborators^16^ provides over 35 million gossip messages collected between 2018 and 2023 using independent C-Lightning nodes. Although this dataset is labeled as “best-effort” and is not guaranteed to be complete, it is distributed under an MIT license and has become a widely recognized and frequently used data source in payment channel network research^3,17^.

For current work, we utilized this public dataset (available at https://github.com/lnresearch/topology)^16^ as our primary data input. Specifically, we downloaded and processed all seven available gossip message snapshots dated 2020-10-14, 2020-11-02, 2020-12-03, 2021-01-04, 2021-09-08, 2022-08-23, and 2023-09-24.

### Stage 2: Network graph reconstruction

Using the downloaded gossip message archives, we reconstructed historical LN states. To achieve this, we adapted and applied TimeMachine^5,16^, an algorithm designed to replay gossip messages up to a specified point in time in order to reconstruct the observed LN topology at that moment. During reconstruction, all messages are deduplicated and chronologically ordered to ensure an accurate replay sequence. The algorithm halts at the target timestamp, yielding a network state that closely approximates the publicly visible LN topology at that date.

We employed TimeMachine to reconstruct the LN topology at two-week intervals corresponding to each gossip message snapshot, beginning from the last available update. Our implementation closely follows the original TimeMachine methodology, with minor modifications to enable seamless reconstruction and storage of biweekly topology slices across all available message archives. Although only seven message snapshots were used, this process generated a substantial dataset of topology slices, approaching near-daily temporal granularity, while accounting for minor technical gaps in message broadcast continuity.

### Stage 3: Pre-processing and consistency control

As described above, we generated at least one corresponding network graph for each two-week interval. During this stage, we examined each graph’s structure and assessed its internal consistency, considering potential overlaps between intervals. In cases of overlap, we retained the version with the highest connectivity (i.e., the most complete and structurally vital graph). We also removed isolated nodes and channels lacking sufficient metadata to ensure that all retained snapshots were consistent and suitable for meaningful analysis.

**Fig. 1:**
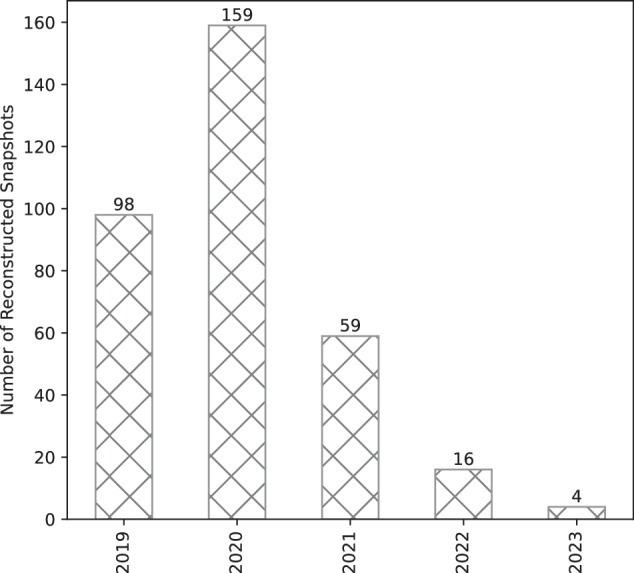
Temporal data coverage.

Following this pre-processing, we obtained 336 consistent network topology snapshots spanning from January 20, 2019, to July 16, 2023, thus covering nearly five years of LN evolution. However, due to the limited availability of input data and the inherent characteristics of the LN, its dynamic nature, partial observability, and the asynchronous propagation of gossip messages, the reconstructed snapshots do not provide uniform temporal coverage, and several gaps remain (Fig. 1).

Figure 1 illustrates the number of reconstructed topology snapshots per year, representing the dataset’s temporal resolution. While these gaps may constrain certain longitudinal analyses of network evolution, imperfect temporal coverage is a common and unavoidable limitation in LN research. Even recent studies have often relied on as few as one or two measurement points per year^18^. By providing a quantitative account of data gaps, easily accessible through the dataset’s metadata, we enable researchers to conduct structural analyses more transparently, incorporate confidence intervals into temporal estimates, or adopt other probabilistic approaches to temporal network analysis rather than relying on manual and arbitrary measurement. Additional details regarding temporal data coverage, limitations, and validation are provided in the technical validation subsection.

### Stage 4: Geolocation

To enrich the dataset with the geographical distribution of LN nodes, we geolocated nodes based on the IP addresses available in the raw input data. At this stage, we extracted both IPv4 and IPv6 addresses and used the IPinfo.io Lite geocoding Application Programming Interface (API, available at https://ipinfo.io) to determine the approximate geographical coordinates of nodes with valid IPs. The retrieved geolocation details were stored in JSON format and integrated into each corresponding node’s metadata within the reconstructed topology snapshots.

The geolocation procedure comprised three major steps:

1. IP Address Extraction and Deduplication
  We extracted all available IP addresses from every snapshot, deduplicated them, and stored them in a key-value storage. This process yielded 7,030 unique and valid IPv4/IPv6 addresses and also identified 11 invalid entries, which were then ignored.
2. Geocoding and Metadata Enrichment
  For each valid IP address, we queried the geocoding API to retrieve approximate geographical coordinates, postal codes, city and region names, and two-letter country codes. When available, the Internet Service Provider (ISP) organization name was also recorded. All valid IPs were successfully supplemented with reliable geolocation data, which were then stored in a structured key-value JSON-like format.
3. Integration and Anonymization
  The obtained geolocation metadata were merged back into the corresponding node entries in each topology snapshot. To comply with data privacy and ethical research policies, all raw IP addresses and hostnames were removed from the final dataset.
  Because the LN supports both public IP and hidden TOR addresses for peer connections (and the latter have become increasingly common due to privacy concerns) the proportion of nodes with publicly visible IP addresses has decreased over time. Consequently, the share of geolocatable nodes in our dataset declines in later snapshots. This proportion is further constrained by limitations in the input data and the accuracy boundaries of the geolocation service used. Thus, while the provided geolocation data offer a meaningful approximation of the LN’s spatial distribution, it is shared without any guarantee of completeness or precision.
  Nonetheless, researchers can replicate or improve these results by modifying our processing pipeline to employ alternative geolocation services (e.g. that improves accuracy based on archival databases) or their own raw data sources. We believe that this dataset enrichment will encourage further research into geolocation challenges and serve as a valuable reference for future analyses of the LN geographic distribution. Additional details regarding spatial data coverage, limitations, and validation are provided in the technical validation subsection.

### Ethics approval & informed consent

Since the data were obtained from publicly available sources and the secondary analysis was performed, informed consent or ethical approval is not required. The dataset does not contain any personally identifiable or sensitive human data. IP-based geolocation was derived using the IPinfo Lite API, which provides approximate city-level coordinates that cannot be used to identify individuals or devices. All IP addresses were removed prior to data sharing, and the released dataset includes only de-identified geographic information.

## Data Records

In this section, we start by describing the data storage, followed by a detailed overview of the dataset’s contents, along with some illustrative examples for reference.

### Data storage

The dataset is publicly available^19^ in the Harvard Dataverse repository and can be accessed at 10.7910/DVN/2OAVO6.

The repository contains geolocated Lightning Network (LN) topology snapshots compressed into a single archive, ‘snapshots.geo.zip’. Each snapshot within the archive is named using the format ‘YYYYMMDD.gml.geo’ (e.g., ‘20190120.gml.geo’), which indicates the corresponding date and file format (GML, Graph Modelling Language). In addition, the dataset includes the readme file and the ‘shapes.geo.csv’ file, which provides metadata for each snapshot.

## Data Overview

This dataset consists of 336 geolocated LN topology snapshots in the GML format, covering the period from 2019 to 2023. Each snapshot represents the LN’s reconstructed network topology at a specific point in time and includes metadata describing node and channel attributes.

The accompanying metadata file, ‘shapes.geo.csv’, provides summary information for each snapshot, including:

- Timestamp and corresponding datetime.
- Number of nodes and channels.
- Average node degree.
- Approximated graph diameter.
- Number of geolocated nodes.
- Associated snapshot file name.

**Fig. 2:**
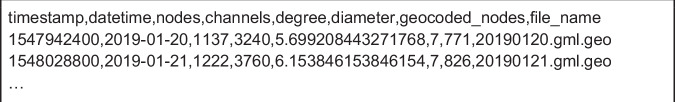
Example content of the snapshot metadata file.

An excerpt of the metadata file is shown in Fig. 2:

Each snapshot can be directly loaded and analyzed using NetworkX^20^, a widely used Python library for network analysis. Snapshots can be imported with the standard function for GML formatted data, e.g. ‘*nx.read_gml*’. The dataset also includes a Python Jupyter Notebook, ‘*example.ipynb*’ demonstrating basic network visualization and analysis techniques using NetworkX.

**Fig. 3:**
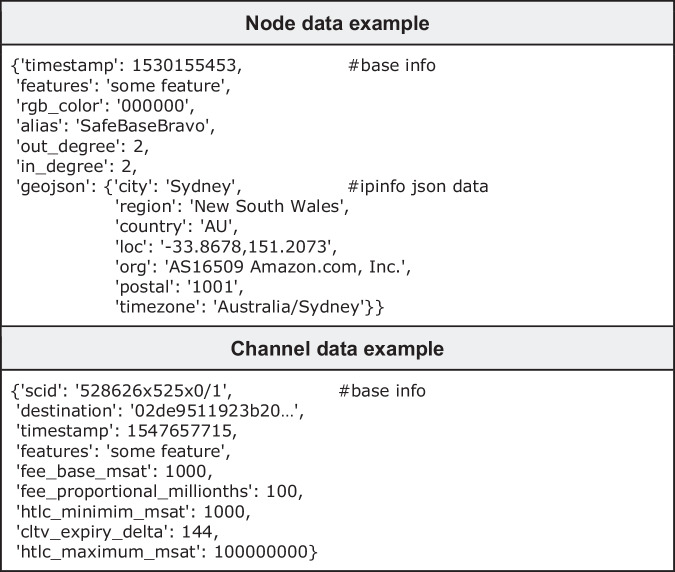
Examples of node and channel data entries.

Within each NetworkX graph object, nodes and channels are represented as Python dictionaries containing relevant attributes and metadata. Figure 3 shows examples of node and channel data entries, including geolocation details.

## Technical Validation and Limitations

The input data used to prepare this dataset were collected from the live LN using a standard node equipped with specialized snapshotting algorithms. Consequently, the dataset’s completeness and accuracy vary over time due to several factors: (1) the dynamic nature of the LN; (2) the asynchronous propagation of gossip messages; and (3) periodic changes in the underlying data collection methodology.

According to the source documentation, certain technical inconsistencies were more frequent in data from early 2018–2019, particularly during transitional periods such as April 2018 and August 2019, see a developer note in^16^. To balance temporal coverage with data quality, we chose January 2019 as the starting point for our cleaned dataset. Despite this refinement, several temporal gaps remain due to the network’s volatility and observational constraints.

### Validation of network size characteristics

To assess dataset validity, we quantitatively analyzed the size characteristics of the reconstructed graphs – specifically, the number of nodes, number of channels, and the approximated graph diameter. These metrics were cross-referenced against independent sources, including BitcoinVisuals (BV, https://bitcoinvisuals.com/lightning) and mempool.space (MP, https://mempool.space/graphs/lightning/nodes-networks).

**Fig. 4:**
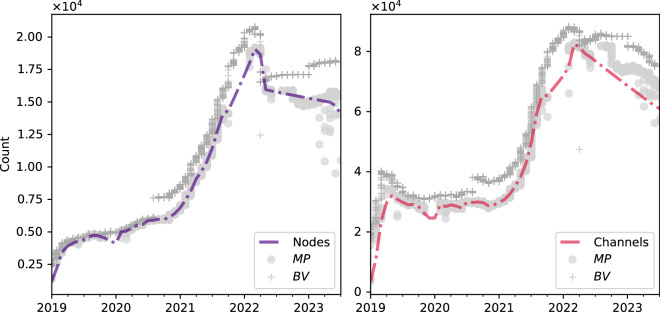
Network size characteristics. *Note*. Monthly averaged size measurements from reference sources (*BV, MP with* dots and crosses, respectively) and our reconstructed snapshots (interpolated dashed lines), *BV* – BitcoinVisuals (https://bitcoinvisuals.com/lightning), *MP* – mempool.space (https://mempool.space/graphs/lightning/nodes-networks).

Figure 4 compares the LN size measurements between 2019 and 2023. The lines represent our dataset’s measurements, while the dots and crosses correspond to BV and MP statistics, respectively.

Because BV and MP gather their data through independent nodes observing different segments of the LN at varying frequencies, some discrepancies are expected – stemming from message propagation latency, node reachability, and measurement timing.

For formal validation, we applied the Kolmogorov–Smirnov (KS) test to evaluate whether our dataset and external sources could be considered samples from the same distribution. As summarized in Table 1, the KS test indicates strong similarity between our dataset and MP statistics, while initially rejecting the null hypothesis for BV data (*p* < 0.05). After removing temporal dependencies (i.e., trend and seasonality effects) from the data, alignment improved, suggesting that the observed differences largely reflect temporal structure rather than systematic bias. This supports the general validity of our reconstructed LN topology snapshots.

**Table 1:** Network size validation.

Size metric	Reference source	Kolmogorov-Smirnov test		Normalized Wasserstein distance	
		*with trend*	*no trend*	*with trend*	*no trend*
Nodes	*MP*	0.091	0.091	0.005	0.025
	*BV*	0.327**	0.145	0.067	0.073
Channels	*MP*	0.091	0.091	0.016	0.053
	*BV*	0.418***	0.091	0.098	0.104
Diameter	*BV*	0.364**	0.182	0.091	0.082

*Note*. The null hypothesis on distributions similarity is tested. *BV* – BitcoinVisuals (https://bitcoinvisuals.com/lightning), *MP* – mempool.space (https://mempool.space/graphs/lightning/nodes-networks), statistical significance: **p < 0.01, ***p < 0.001, “*no trend*” denotes removing either trend or seasonal components.

To further quantify distributional similarity, we computed the normalized Wasserstein distance (WD), which measures the minimal “cost” of transforming one distribution into another. A smaller WD indicates greater similarity. For interpretability, WD values were normalized by dividing by the maximum observed values (nodes, channels, and diameter). As shown in Table 1, in the worst-case scenario, approximately 10.4% of channels and 7.3% of nodes would need to be redistributed across snapshots to achieve full alignment with reference statistics, which we consider acceptable.

Overall, BV and MP reference series are statistically close to the dynamics captured in our dataset, especially during periods of heightened LN activity. Although some deviations persist, they are expected given the independent observation methods and sampling limitations. This degree of alignment provides confidence in the reliability and representativeness of the reconstructed dataset.

### Validation of spatial coverage

To assess geolocation quality, we examined the proportion of geolocated nodes and compared our results with mempool.space statistics (MP, https://mempool.space/graphs/lightning/nodes-networks) on the share of LN nodes disclosing public IP addresses. According to MP, approximately 41% of nodes had open IPs as of January 1, 2021, decreasing to about 15% by January 1, 2023. This decline reflects the well-documented shift toward privacy-preserving TOR connections. Our dataset exhibits a similar trend, with the average share of 39% ± 15% throughout 2019–2023, indicating acceptable coverage consistent with observed behavior.

To further benchmark our geolocation accuracy, we compared our results to country-level node distribution data reported in an independent study^21^. Because the exact measurement timestamp was not disclosed, we selected the snapshot from our dataset that most closely matched the reported network size (12,359 nodes) and the publication date. Table 2 presents the reference and our proportions, along with the corresponding differences.

**Table 2:** Geolocation accuracy validation.

Country	Reference values, %	Our snapshot estimates, %	Difference
US	10.53	9.59	0.94
FR	5.71	4.26	1.45
DE	3.32	3.62	−0.30
CA	2.49	2.51	−0.02
NL	1.38	1.29	0.09
GB	1.18	1.14	0.04
CN	0.59	0.83	−0.24
IT	0.38	0.36	0.02
JP	0.37	0.36	0.01
Others	5.30	4.99	0.31

*Note*. The reference values obtained from^21^, our snapshot dated at 01.08.2021 consists of 12,089 nodes (776 geolocated nodes).

**Fig. 5:**
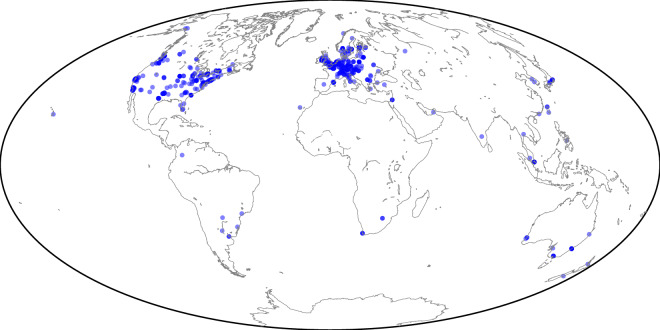
Geographical node distribution. *Note*. The snapshot dated at 01.08.2021 consists of 12,089 nodes (776 geolocated nodes).

The reference study reported a share of nodes had open IPs of 31.3%, while our matching snapshot indicated 28.9%. Although country-level differences are observable (Table 2), the overall geographical distribution (Fig. 5) remains consistent, with a root mean square deviation (RMSD) for geolocation accuracy of 0.57% – an acceptable margin given the dynamic and privacy-oriented nature of the LN. The Spearman correlation coefficient between country-level reference values and our snapshot estimates is 0.98, with *p* < 0.0001.

Although IP-based geolocation is inherently imprecise, particularly for privacy-enhanced networks such as LN, we successfully obtained geolocation information for all 7,030 valid IP records. However, only a matching snapshot containing 776 geolocated nodes (Fig. 5) was validated against the sole available independent country-level reference study^21^. To our knowledge, few prior studies have attempted to analyze the geographical distribution of LN nodes at this scale. We hope this dataset encourages further research and provides a solid empirical reference for future work in this area.

### Limitations and future directions

The dataset inevitably reflects several methodological and observational limitations:

- Temporal gaps. The reconstructed snapshots exhibit irregular time intervals, restricting fine-grained analyses of specific historical events in the LN’s evolution. These discontinuities may influence longitudinal inferences but also highlight opportunities for improving sampling methods and interpolation techniques.
- Geolocation accuracy. The reliance on IP-based geolocation introduces uncertainty, particularly for TOR-based or VPN-obscured nodes. This may bias geographic analyses or underrepresent regions with stronger privacy adoption.
- Reliability and validation. Both internal and external reference datasets and statistics are “best-effort” in nature, meaning exact alignment is neither possible nor expected. Nevertheless, our cross-validation confirms a reasonable degree of alignment.

Recognizing these challenges provides a valuable foundation for methodological refinement. Future research could integrate multiple independent data sources, apply imputation or temporal modeling to fill observational gaps, or explore hybrid geolocation techniques that balance accuracy with privacy preservation.

In summary, despite inherent constraints, the dataset demonstrates strong statistical consistency with independent measurements and offers a validated benchmark for studying LN structure, dynamics, and geography. We hope that this transparent documentation of limitations further enhances its credibility and utility for the broader research community.

## Data Availability

The dataset is publicly available^19^ in the Harvard Dataverse repository and can be accessed at 10.7910/DVN/2OAVO6.

## Code availability

The code is available in the GitHub repository https://github.com/ellariel/ln-data-preparation and has been shared alongside the dataset. This repository contains Python scripts for the data extraction and transformation pipelines, as well as all code necessary to reproduce the figures and calculations presented in this paper.

## References

[1] Papadis, N. & Tassiulas, L. Blockchain-based payment channel networks: challenges and recent advances. *IEEE Access* **8**, 227596–227609, 10.1109/ACCESS.2020.3046020 (2020).

[2] Valko, D. & Marx Gómez, J. Recent advances in global payment channel networks: a systematic literature review. *Res. Sq*. **preprint**, 10.21203/rs.3.rs-7705514/v1 (2025).

[3] Zabka, P., Foerster, K.-T., Schmid, S. & Decker, C. Empirical evaluation of nodes and channels of the Lightning Network. *Pervasive Mob. Comput.* **83**, 101584, 10.1016/j.pmcj.2022.101584 (2022).

[4] Khamis, J., Kotzer, A. & Rottenstreich, O. Topologies for blockchain payment channel networks: models and constructions. *IEEE/ACM Trans. Netw.* **32**, 4781–4797, 10.1109/TNET.2024.3445274 (2024).

[5] Zabka, P., Förster, K.-T., Decker, C. & Schmid, S. A centrality analysis of the Lightning Network. *Telecommun. Policy* **48**, 102696, 10.1016/j.telpol.2023.102696 (2024).

[6] Kappos, G. *et al*. An empirical analysis of privacy in the Lightning Network. In Borisov, N. & Diaz, C. (eds) *Financial Cryptography and Data Security – 25th International Conference, FC 2021, Revised Selected Papers. Lect. Notes Comput. Sci*. **12674**, 167–186, 10.1007/978-3-662-64322-8_8 (Springer, 2021).

[7] Mizrahi, A. & Zohar, A. Congestion attacks in payment channel networks. In Borisov, N. & Diaz, C. (eds) *Financial Cryptography and Data Security – 25th International Conference, FC 2021, Revised Selected Papers. Lect. Notes Comput. Sci*. **12675**, 170–188, 10.1007/978-3-662-64331-0_9 (Springer, 2021).

[8] Rohrer, E., Malliaris, J. & Tschorsch, F. Discharged payment channels: quantifying the Lightning Network’s resilience to topology-based attacks. In *2019 IEEE European Symposium on Security and Privacy Workshops (EuroS&PW)*, 347–356, 10.1109/EuroSPW.2019.00045 (IEEE, 2019).

[9] Corcoran, P. & Lewis, R. *An analysis of the correctness and computational complexity of path planning in payment channel networks*. *arXiv***preprint: 2501.11419**, 10.48550/arXiv.2501.11419 (2025).

[10] Guo, Y., Tong, J. & Feng, C. A measurement study of Bitcoin Lightning Network. In *Proceedings of the 2019 2nd IEEE International Conference on Blockchain (Blockchain 2019)*, 202–211, 10.1109/Blockchain.2019.00034 (IEEE, 2019).

[11] Saraswathi, S. & Kümmerle, C. An exposition of pathfinding strategies within Lightning Network clients. In *Proceedings of the IEEE International Conference on Blockchain and Cryptocurrency (ICBC)*, 1-17, 10.1109/ICBC64466.2025.11114515 (IEEE, 2025).

[12] Papadis, N. & Tassiulas, L. Deep reinforcement learning-based rebalancing policies for profit maximization of relay nodes in payment channel networks. In Pardalos, P., Kotsireas, I., Knottenbelt, W. J. & Leonardos, S. (eds) *Mathematical Research for Blockchain Economy. MARBLE 2023. Lecture Notes in Operations Research*, 1–27, 10.1007/978-3-031-48731-6_1 (Springer, 2023).

[13] Camilo, G. F. *et al*. Topological evolution analysis of payment channels in the Lightning Network. In *2022 IEEE Latin-American Conference on Communications (LATINCOM)*, 1–6, 10.1109/LATINCOM56090.2022.10000445 (IEEE, 2022).

[14] Divakaruni, A. & Zimmerman, P. The Lightning Network: turning Bitcoin into money. *Finance Res. Lett.* **52**, 103480, 10.1016/j.frl.2022.103480 (2023).

[15] dos Santos, S. *et al*. On the impact of the Lightning Network on Bitcoin transaction fees and network value. In *Proceedings of the 2024 IEEE International Conference on Blockchain (Blockchain)*, 148–156, 10.1109/Blockchain62396.2024.00028 (IEEE, 2024).

[16] Decker, C. Lightning Network research — topology datasets [Dataset]. *Zenodo*10.5281/zenodo.4088530 (2020).

[17] Zabka, P., Förster, K.-T., Schmid, S. & Decker, C. Node classification and geographical analysis of the Lightning cryptocurrency network. In *Proceedings of the 22nd International Conference on Distributed Computing and Networking (ICDCN ’21)*, 126–135, 10.1145/3427796.3427837 (Association for Computing Machinery, 2021).

[18] Atmanavičiūtė, L., Vanagas, T. & Masteika, S. Quantitative analysis of centralization in the Bitcoin Lightning Network through centrality metrics. *IEEE Access* **13**, 168761–168781, 10.1109/ACCESS.2025.3614085 (2025).

[19] Valko, D. & Marx Gómez, J. Geolocated Lightning Network topology snapshots: a dataset covering 2019–2023. *Harvard Dataverse*10.7910/DVN/2OAVO6 (2025). 10.1038/s41597-025-06413-7 PMC12698858 41372243

[20] Hagberg, A. A., Schult, D. A. & Swart, P. J. Exploring network structure, dynamics, and function using NetworkX. In Varoquaux, G., Vaught, T. & Millman, J. (eds) *Proceedings of the 7th Python in Science Conference (SciPy2008)*, 11–15, https://networkx.org/documentation/stable/index.html (Pasadena, CA, USA, 2008).

[21] Howell, A., Saber, T. & Bendechache, M. Measuring node decentralisation in blockchain peer-to-peer networks. *Blockchain: Res. Appl.* **4**, 100109, 10.1016/j.bcra.2022.100109 (2023).

